# Endoscopic Resection of an Esophageal Gastrointestinal Stromal Tumor After Neoadjuvant Treatment: A New Paradigm for Minimally Invasive Therapy?

**DOI:** 10.14309/crj.0000000000001901

**Published:** 2025-11-24

**Authors:** Fabian Dario Rodriguez-Monaco, Maximilian Herter, Dolores Krauss, Hans Fuchs, Tomasz Zienkiewicz, Franz Ludwig Dumoulin

**Affiliations:** 1 Department of Medicine/Gastroenterology, Gemeinschaftskrankenhaus Bonn, Bonn, Germany; 2 Department for General, Visceral, Thoracic and Transplantation Surgery, Faculty of Medicine, University Hospital Cologne, University of Cologne, Cologne, Germany; 3 MVZ for Pathology/Molecular Pathology Bonn, Bonn, Germany

**Keywords:** gastrointestinal stromal tumor, GIST, neoadjuvant therapy, submucosal tunnel endoscopic resection, STER

## Abstract

Esophageal gastrointestinal stromal tumors (eGISTs) are rare. Current guidelines recommend surgical R0 resection. Here, we report on a patient diagnosed with a larger (>30 mm) localized GIST in the midesophagus. After interdisciplinary discussion and discussion with the patient, we initiated a neoadjuvant therapy with imatinib 400 mg. The tumor was downsized and removed uneventfully by endoscopic tunneling resection. Histopathology showed a 19 × 18 × 17 mm ypT1-R1 eGIST. After shared decision-making, the patient continues therapy with imatinib and is in remission 1 year after resection. Downsizing by neoadjuvant treatment could increase the number of endoscopically resectable eGISTs.

## INTRODUCTION

Gastrointestinal stromal tumors (GIST) are mesenchymal tumors originating from interstitial cells of Cajal. Tumor site, size, mitotic count, and mutational analysis are of prognostic importance and guide further management.^1^ Complete (R0) surgical resection without lymphadenectomy is the standard treatment for localized GIST > 20 mm. Neoadjuvant kinase inhibitor treatment can be considered in selected cases.^2^ In addition, an R1 resection (involved margins but intact tumor capsule) for GISTs is accepted, e.g., after resection in unfavorable localization. Adjuvant treatment for 3 years is recommended in cases with high risk for relapse.^3^

Although GISTs most commonly occur in the stomach, esophageal localization (eGIST) accounts for less than 1% of all GIST cases.^1^ Until recently, the standard treatment for eGIST has been surgical resection, with the specific approach determined by tumor size and localization.^4,5^ Given the excellent efficacy of kinase inhibitors, there are also case reports of neoadjuvant treatment of larger eGISTs, followed by surgical resection.^5–8^ Endoscopic resection techniques, particularly so-called “third-space endoscopy,” have shown some promising results for nonsurgical treatment of eGIST. Thus, a recent case series on 31 patients reported an R0 resection rate of 75% of mostly very low or low risk eGISTs with a low complication rate.^9^ Another series of 22 patients undergoing endoscopic resection reported similar findings, with an R0 rate of 78%.^10^ Here, we present a case report of a patient who underwent successful endoscopic resection of an eGIST after neoadjuvant therapy with imatinib.

## CASE REPORT

**Figure 1.**
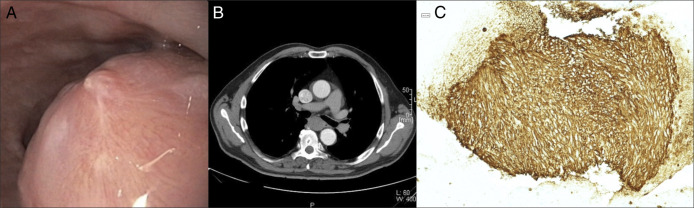
Primary diagnostic workup. (A) Endoscopic appearance of the subepithelial lesion in the mid esophagus. (B) Computed tomography scan illustrating the localization at the level of the tracheal bifurcation. (C) Immunohistochemistry (DOG-1) of endoscopic ultrasound and fine-needle aspiration cytology specimen demonstrating a gastrointestinal stromal tumors—with a low mitotic index.

**Figure 2.**
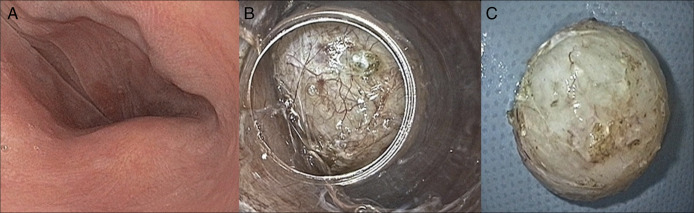
Submucosal endoscopic tunnel resection after 11 months of neoadjuvant therapy with imatinib. (A) Endoscopic aspect of the downsized lesion after neoadjuvant therapy. (B) Submucosal tunnel entrance was created at 18 cm from incisors with a tunnel length of approximately 5 cm; creation of the tunnel was smooth without any bleeding during the preparation. The picture shows the situation where dissection of the gastrointestinal stromal tumors was nearly completed. The tunnel entrance was subsequently closed with 4 hemoclips. (C) Resected specimen, about 20 mm.

**Figure 3.**
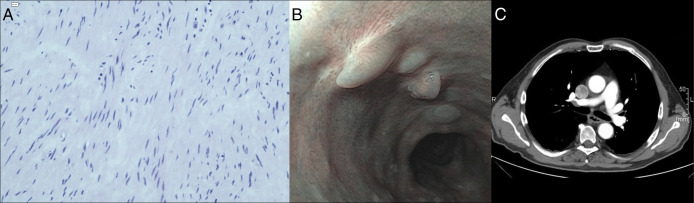
Results after endoscopic resection. (A) Histopathology of the resected specimen (hematoxylin & eosin, 200×). (B) Endoscopic aspect of the scar at the site of the former tunnel entry. The 4 small nodules are hypertrophic scars at the clip closure site of the former tunnel entrance. (C) Computed tomography scan confirming complete remission.

A 68-year-old gentleman without relevant comorbidity presented with intermittent dysphagia. Subsequent workup with endoscopy, endoscopic ultrasound (EUS), and fine-needle aspiration cytology revealed a subepithelial lesion of > 30 mm in the midesophagus at the site of the tracheal bifurcation without significant stenosis. EUS showed a well-demarcated homogeneous hypoechoic lesion located in a proper muscle layer. The staging computed tomography (CT) did not show any metastasis. EUS-fine-needle aspiration established the diagnosis of a localized GIST with a mitotic index of approximately 1% and a *KIT* exon 11 mutation (Figure 1). The patient was deemed fit for surgery but actively asked for an organ preserving treatment. All results of the workup were discussed in the interdisciplinary tumor board. Given the molecular susceptibility of the eGIST, it was consented to offer the patient a neoadjuvant treatment with imatinib to downsize the lesion an facilitate later endoscopic resection. After a further detailed discussion with the patient and his daughter, a neoadjuvant treatment with daily imatinib 400 mg was initiated under endoscopic and CT monitoring every 3–4 months. Under treatment, there were no further bouts of severe dysphagia; the patient did not lose any weight. After 11 months of treatment, the lesion size had regressed to ca. 2 cm. The lesion was then removed by submucosal tunneling endoscopic resection with an uneventful postprocedural course (Figure 2). Histopathologic analysis showed a GIST measuring 19 × 18 × 17 mm, staged as ypT1-R1 with a mitotic rate of < 1 per 50 high-power field and scored as very low risk of metastasis according to the Armed Forces Institute of Pathology risk classification. After re-evaluation in interdisciplinary tumor board, continuation of imatinib was recommended. Follow-up after 1 year confirmed remission (Figure 3). We plan to continue endoscopic controls every 3 months and CT scans every 6 months for the first 2 years and yearly endoscopic controls thereafter for up to 5 years.

## DISCUSSION

EGISTs account for less than 1% of all GISTs and therefore evidence for prognosis, and treatment is very limited. EGISTs are believed to carry a worse prognosis than gastric GISTs.^1^ Current guidelines recommend an R0 resection without lymph node dissection for lesions larger than 20 mm.^11^ However, surgical resection of eGISTs is burdened by relevant complication rates. Thus, a surgical case series with 50 patients reported adverse events >/= Clavien-Dindo > 3b in over 16%.^4^ With the advent of third space endoscopy, endoscopic resection for eGIST has gained some interest. A case series with 32 patients with a mean tumor size of 2.21 cm showed en bloc and R0 rates of 97% and 75%. The complication rate was relatively high (25%), but the severity was relatively modest (hydrothorax and postendoscopic coagulation syndrome all of which could be managed conservatively).^9^ More important, the prognosis was favorable with a 5-year overall survival of 100% and recurrence-free survival of 91%, respectively. Another series with 22 cases suggested feasibility of endoscopic resection of eGISTs with sizes of up to 5 cm.^10^ Although neoadjuvant therapy of large eGISTs with consecutive surgery has been described, we are not aware of reports on neoadjuvant treatment to downsize eGISTs to facilitate subsequent endoscopic resection.^5–8^ In the case reported here, the tumor size had regressed to ca. 20 mm after 11 months of imatinib therapy. The lesion was removed endoscopically without complications. The histopathology revealed a low-risk GIST, ypT1-R1.

In combination with the data from Xu et al, it could be postulated that endoscopic resection is relatively safe for this tumor size.^9^ Therefore, neoadjuvant downsizing could not only increase general endoscopic resectability but also has the potential to reduce complication rates. This hypothesis is supported by data from the National Cancer Database that demonstrated a slight benefit of neoadjuvant treatment both for overall survival (hazard ratio 0.85) and 90-day postoperative mortality (0.5% vs 2.2%) in 16,308 surgically treated GIST patients analyzed.^6^ It has however to be kept in mind that escape mutations can occur under imatinib treatment, so prolonged neoadjuvant treatment should probably be avoided.^12^ The decision to continue imatinib as adjuvant treatment was not based on the R1 resection status. In fact, a large surgical study on 906 patients with mainly gastric and small bowel GISTs showed that an R1 resection in the absence of capsule rupture did not result in worse outcome during a follow-up of more than 9 years.^13^ Still in this particular situation, we offered continuation of imatinib which had been very well tolerated after shared decision-making with the patient.

Although we think our case report highlights the possibility for neoadjuvant treatment of eGIST—with downsizing and subsequent endoscopic resection—we do have to acknowledge several weaknesses and caveats. Thus, the follow-up period of 1 year is relative short for a low-risk GIST. Moreover, there are no data on the significance of an R1 resection for eGISTs after neoadjuvant therapy.

In summary, our case report demonstrates that neoadjuvant downsizing could be helpful to facilitate subsequent endoscopic resection. Given an appropriate genetic mutation, the efficacy of imatinib in esophageal GISTs is well established.^2–5^ The neoadjuvant use of tyrosine kinase inhibitors may allow a greater proportion of patients to undergo organ-preserving treatment.

## DISCLOSURES

Author contributions: FD Rodriguez-Monaco provided the first draft of the manuscript. FD Rodriguez-Monaco and M. Herter designed the endoscopic figures. T. Zienkiewicz contributed histopathology figures. All authors provided clinical data and data from the literature for this report. All authors worked on the revisions of earlier drafts and reviewed and approved the first submission as well as the revised version of the manuscript. FL Dumoulin is the article guarantor.

Financial disclosure: None to report.

Previous presentation: This case was previously presented as an oral presentation at the DGE-BV meeting, Würzburg, Germany, March 20, 2025.

Informed consent was obtained for this case report.

## ABBREVIATIONS:

CT: computed tomography
eGIST: esophageal gastrointestinal stromal tumor
EUS: endoscopic ultrasound
GIST: gastrointestinal stromal tumor
